# Sustainable citric acid production from CO_2_ in an engineered cyanobacterium

**DOI:** 10.3389/fmicb.2022.973244

**Published:** 2022-08-17

**Authors:** Lifang Zhang, Samantha J. Bryan, Tiago Toscano Selão

**Affiliations:** Department of Chemical and Environmental Engineering, University of Nottingham, Nottingham, United Kingdom

**Keywords:** citrate, cyanobacteria, sustainable production, riboswitch, photosynthesis

## Abstract

Citric acid is one of the most widely used organic acids in the world, with applications ranging from acidity regulation in food and beverages to metal chelation in hydrometallurgical processes. Most of its production is currently derived from fermentative processes, using plant-derived carbon feedstocks. While these are currently dominant, there is an increasing need to develop closed-loop production systems that reduce process carbon footprint. In this work, we demonstrate for the first time that an engineered marine cyanobacterium *Synechococcus* sp. PCC 7002 can be used as a sustainable chassis for the photosynthetic conversion of CO_2_ to citric acid. Decreased citric acid cycle flux, through the use of a theophylline-responsive riboswitch, was combined with improved flux through citrate synthase and enhanced citric acid excretion, resulting in a significant improvement to citric acid production. While allowing citrate production, this strategy induces a growth defect which can be overcome by glutamate supplementation or by fine-tuning aconitase levels, resulting in an increase in production relative to WT of over 100-fold. This work represents a first step toward sustainable production of a commodity organic acid from CO_2_.

## Introduction

Atmospheric CO_2_ concentration in 2021 surpassed 400 ppm, a new record high (Rebecca, 2018; Li et al., 2022). This increase is linked to changes in weather patterns, leading to extreme weather events. At the same time, an increasing human population, which is often linked to deforestation and freshwater scarcity, exacerbates the pressure on natural ecosystems. Solving these pressing issues requires holistic novel production routes that not only circumvent the usage of natural resources but also have either a low or negative carbon footprint.

Biotechnology has already demonstrated the capacity to respond to some of these challenges, by generating microbial strains able to produce vital compounds for our everyday lives from renewable carbon feedstocks (glucose or glycerol). An example of this can be found in the engineering of the yeast *Yarrowia lipolytica* to convert glucose into the biofuel precursors fatty acid methyl esters, with productivities reaching 1.2 g/L/h (Qiao et al., 2017) Another example of the power of biotechnology comes from the utilization of engineered *Aspergillus niger* to produce citric acid, a process that generates 1.9 million tons of this organic acid per year (Max et al., 2010; Kirimura et al., 2011). Citric acid is one of the most widely produced organic acids in the world and is used in a wide range of applications, from food and beverage additives to pharmaceutical ingredients and in hydrometallurgical battery recycling processes (Behera et al., 2021). Biologically, citric acid synthesis is catalyzed by a single group of enzymes, citric acid synthases, using acetyl-CoA and oxaloacetate as substrates (Wiegand and Remington, 1986; Duckworth et al., 1987). This first committed catalytic reaction of the tricarboxylic acid (TCA) cycle is therefore highly regulated, especially at the post-translational level. This regulation differs between phyla, with Gram-negative bacterial enzymes being inhibited by NADH while Gram-positive are not (Behera et al., 2021). It was recently demonstrated that overexpression of a citric acid exporter gene in *A. niger* increased productivity fivefold to 109 g/L (Steiger et al., 2019). Furthermore, this was transferable to other organisms, such as *Saccharomyces cerevisiae* [where CexA overexpression allowed the accumulation of 0.3 g/L of citric acid in the growth medium vs. no measurable production for the WT (Steiger et al., 2019)], and other filamentous fungi, increasing citric acid accumulation 3.2-fold for *Aspergillus kawachii* (to 0.4 g/g dry cell weight) and 220-fold (to 0.16 g/g dry cell weight) for *Aspergillus oryzae* CexA overexpression strains (Nakamura et al., 2021).Thus, it is possible that CexA overexpression would benefit other engineered citric acid-producing microorganisms.

**Figure 1:**
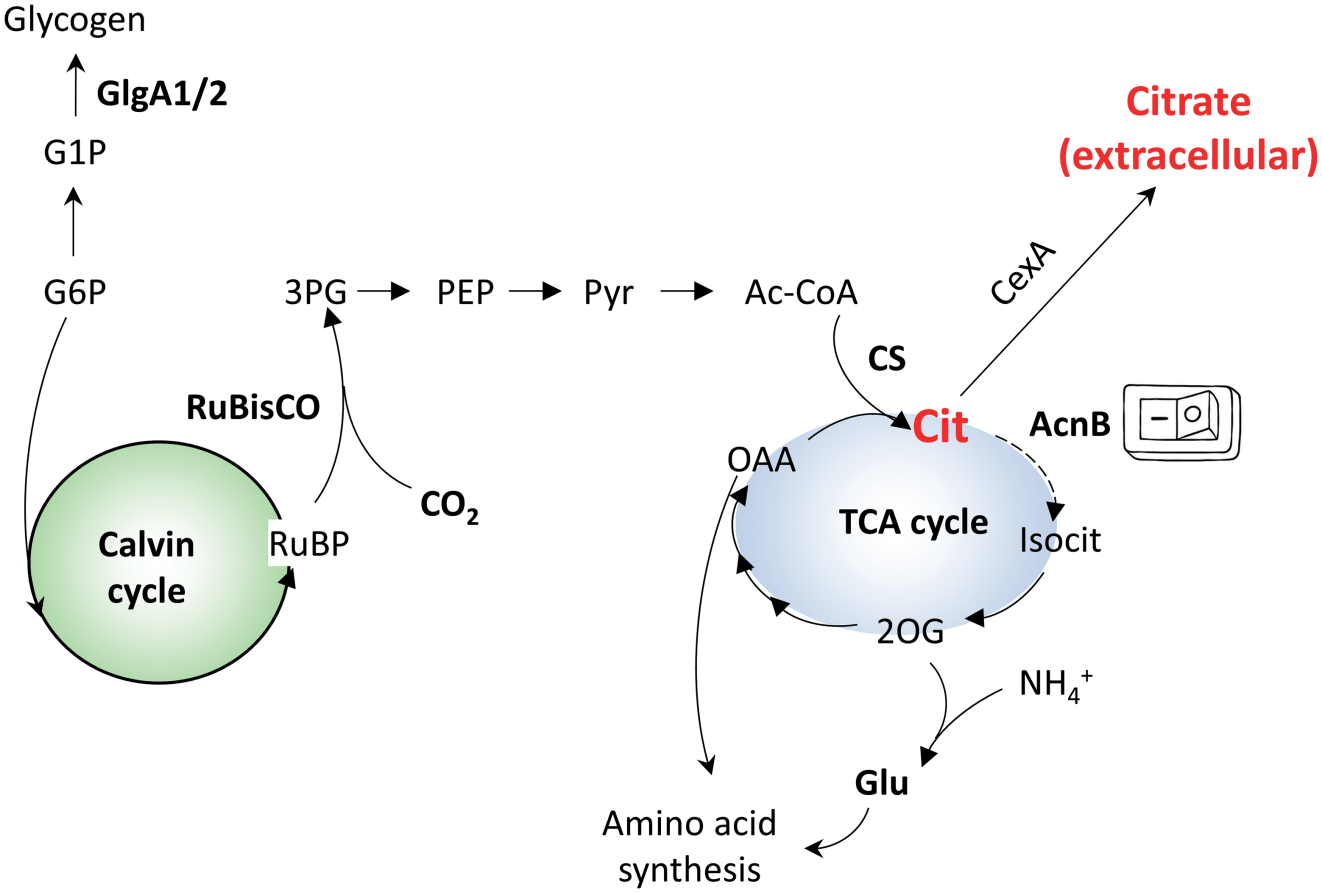
Simplified overview of the central metabolism of Syn7002. G1P - glucose-1-phosphate; G6P - glucose-6-phosphate; RuBP - ribulose bisphosphate; 3PG - 3-phosphoglycerate; PEP - phosphoenolpyruvate; Pyr - pyruvate; Ac-CoA - acetyl-CoA; OAA - oxaloacetate; 2OG - 2-oxoglutarate; Isocit - isocitrate; Cit - citrate; CS - citrate synthase; Glu - glutamate.

Cyanobacteria are able to convert CO_2_ from, e.g., flue gases into target compounds, providing a sustainable alternative to bioproduction. In particular, euryhaline strains, such as *Synechococcus* sp. PCC 7002 (Syn7002) or *Synechococcus* sp. PCC 11901, able to grow on seawater with minimal nutrient input and deriving their energy from sunlight, can be used to close the production loop and reduce the use of arable land (required for production of carbon sources to use in fermentative bioprocesses). The recent characterization of robust, fast-growing species (Włodarczyk et al., 2020) as well as the demonstration that high-density cultivation can substantially improve target compound production (Włodarczyk et al., 2020) demonstrate their potential as chassis for renewable biotechnology. In parallel, the development of advanced synthetic biology tools for cyanobacteria, including promoter toolboxes and riboswitches (Chi et al., 2019), also contributes toward their adoption in biotechnology processes. Cyanobacteria have an unusual TCA cycle, lacking the enzyme 2-oxoglutarate dehydrogenase, which is functionally substituted by the enzymes 2-oxoglutarate decarboxylase and succinic semialdehyde dehydrogenase (Steinhauser et al., 2012; Figure 1). This unusual TCA cycle may be an evolutionary adaptation of cyanobacteria to their particular lifestyle, facilitating the production of biosynthetic intermediates rather than reducing equivalents, as these are directly derived from the photosynthetic linear electron flow (Steinhauser et al., 2012).

In this work, we have engineered the euryhaline cyanobacterium *Synechococcus* sp. PCC 7002 for production of citric acid from CO_2_ and demonstrate that modulating the metabolic flux through the TCA cycle is critical toward accumulating enough citric acid for export. Moreover, our results also point to substantially different cellular requirements for flux through the TCA cycle in low-density vs. high-density cultivation conditions. Our work shows that, while volumetric production is currently low, citric acid production from CO_2_ using engineered cyanobacteria is possible, paving the way for closed-loop production systems.

## Materials and methods

### Strains, media, and growth conditions

Syn7002 strains were routinely grown in either AD7 or MAD medium, as previously described (Włodarczyk et al., 2020). All experiments were performed at 30°C and strains were grown in a Fitotron growth chamber (Weiss Technik), under continuous white LED illumination, at either 50 μmol photons·m^−2^·s^−1^ and ambient air or 300 μmol photons·m^−2^·s^−1^ and 1.5% CO_2_ (v/v)-enriched air (using a custom-made airtight enclosure).

Growth and performance of the different strains were tested in 15 ml cultures, grown in upright tissue culture flasks (Corning, part #3056), inoculated at a starting OD_730_ of 0.1, and measured in a 1 cm-light path UV mini1240 spectrophotometer (Shimadzu) using AD7 as blank. Evaporative water loss was estimated by average weight loss across culture vessels and compensated by daily addition of corresponding volume of sterile MQ water. Whole-cell spectra (between 400 and 730 nm) were recorded in a UV2600i spectrophotometer (Shimadzu), as described above.

All cloning steps were performed using *Escherichia coli* Stellar supercompetent cells (TaKaRa), grown in Luria-Bertani (LB) medium supplemented with the appropriate antibiotics, as indicated.

### Cloning and cyanobacterial transformation

Plasmids generated in this work (see Table 1) were constructed using the Q5 High Fidelity 2x Master Mix and NEBuilder HiFi DNA assembly (NEB), according to the manufacturer’s instructions, and transformed into supercompetent *E. coli* Stellar cells. For a list of all employed primers please consult Supplementary Table S1. Syn7002 transformation was performed as previously described (Selão et al., 2019). Strains were selected in AD7 agar plates [supplemented with 1.2% (w/v) agar and 1 g·L^−1^ sodium thiosulfate prior to autoclaving] using antibiotics as described (50 μg·ml^−1^ spectinomycin, 10 μg·ml^−1^ chloramphenicol, or 100 μg·ml^−1^ kanamycin or combinations thereof) and 2 mM theophylline, for constructs including the P_trc_-E* promoter-riboswitch, unless otherwise specified. All plasmid sequences were confirmed by Sanger sequencing and full genomic segregation was tested by colony PCR using specific primers (see Supplementary Figure S1). The *acnB* locus was amplified from selected strains (for Sanger sequencing) using Q5 High Fidelity 2x Master Mix and the specific *acnB_F* and *acnB_R* primers (Supplementary Figure S1).

**Table 1:** Plasmids generated in this work.

Plasmid name	Plasmid characteristics
pTS019	pUC19-Δ*glgA1*::P_A2579_ − *_ec_gltA*-FLAG − TL3S2P21 − CmR
pTS020	pUC19-Δ*glgA1*::P_A2579_ − _6803_*gltA*-FLAG − TL3S2P21 − CmR
pTS021	pUC19-Δ*glgA1*::P_A2579_ − *_vn_gltA*-FLAG − TL3S2P21 − CmR
pTS022	pUC19-Δ*glgA1*::P_A2579_ − *_sc_gltA*-FLAG − TL3S2P21 − CmR
pTS028	pUC19-KanR − P_trc_-E* − *acnB*
pTS034	pUC19-Δ*glgA1*::P_A2579_ – _6803_*gltA*-FLAG − *_an_cexA* − TL3S2P21 − *CmR*
pTS035	pUC19-Δ*glgA1*::P_A2579_ − *_vn_gltA*-FLAG − *_an_cexA* − TL3S2P21 − *CmR*

Plasmids overexpressing different citrate synthases were based on the pBS07 plasmid (Zhang et al., 2019), targeting the *glgA1* locus of Syn7002. This plasmid was reverse PCR amplified using primers ICA_*glgA1*_CmR_open_F and ICA_*glgA1*_CmR_open_R and used as backbone for overexpression. Citrate synthase genes were amplified from *E. coli* Stellar, *V. natriegens* DSM 759, *Synechocystis* sp. PCC 6803 (Syn6803), or *Streptomyces coelicolor* genomic DNA, using primers indicated in Supplementary Table S1. The strong constitutive promoter of the A2579 gene (Ruffing et al., 2016) was amplified from Syn7002 WT cells using primers Gb_PA2579_F and Gb_PA2579_R and used to generate plasmids pTS019, pTS020, pTS021, and pTS022 (for A2579-based overexpression of *E. coli, V. natriegens*, Syn6803, or *S. coelicolor* citrate synthases respectively, from the *glgA1* locus of Syn7002).

Plasmids pTS020 and pTS021 were opened using primers Gb_Open_CS_F and Gb_Open_CS_R and the *cexA* citrate exporter gene from *A. niger* was amplified from plasmid pMST-1,312 (a gift from Michael Sauer, Addgene plasmid # 118075), with the resulting DNA fragments used to generate plasmids pTS034 and pTS035, respectively.

For control of AcnB expression in Syn7002, a region 500 bp up-and downstream of the *acnB* gene start codon was amplified from Syn7002 genomic DNA and inserted into a PCR-amplified pUC19 backbone, resulting in plasmid pTS026. This plasmid was reverse PCR-amplified using primers Gb_open_*acnB*_F and Gb_open_*acnB*_R and combined with both a kanamycin cassette, amplified from pSZT025 (Włodarczyk et al., 2020), and a synthetic dsDNA fragment containing the previously described P_trc_-E* promoter-riboswitch combination (Ma et al., 2014), resulting in pTS028.

**Figure 2:**
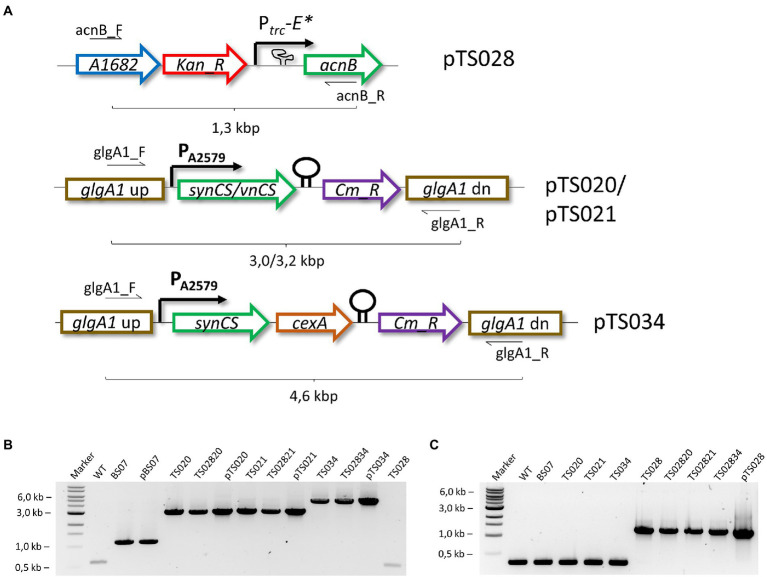
Generation of citric acid producing strains. **(A)** Simplified overview of modified Syn7002 loci for citrate production. *Kan_R* – kanamycin resistance cassette; *synCS* – Synechocystis sp. PCC 6803 citrate synthase gene; *vnCS* – *Vibrio natriegens* citrate synthase gene; *Cm_R* – chloramphenicol resistance cassette. **(B)** Genomic DNA PCR analysis of generated strains, using glgA1_F and glgA1_R primers. **(C)** Genomic DNA PCR analysis of generated strains, using acnB_F and acnB_R primers.

All plasmids were used to transform Syn7002 WT, as described above, generating the corresponding strains (Table 2). Once full segregation was confirmed for strains TS020, TS021, TS034, and TS035 (see Figures 2B,C), these background strains were transformed with pTS028, to generate the corresponding second-or third-generation citrate-producing strains (see Table 2).

**Table 2:** Strains used or generated in this work.

Strains	Genotype
WT	*Synechococcus* sp. PCC 7002
BS07	Δ*glgA1*::CmR (Zhang et al., 2019)
TS020	Δ*glgA1*::P_A2579_ − _6803_*gltA*-FLAG − TL3S2P21 − CmR
TS021	Δ*glgA1*::P_A2579_ − *_vn_gltA*-FLAG − TL3S2P21 − CmR
TS028	KanR − P_trc_-E* − *acnB*
TS034	Δ*glgA1*::P_A2579_ − _6803_*gltA*-FLAG − *_an_cexA* − TL3S2P21 − CmR
TS035	Δ*glgA1*::P_A2579_ − *_vn_gltA*-FLAG − *_an_cexA* − TL3S2P21 − CmR
TS02820	TS020 + KmR − P_trc_-E* − *acnB*
TS02834	TS034 + KmR − P_trc_-E* − *acnB*

### Citric acid concentration measurements

All citric acid-producing cultures were cultured in either AD7 or MAD medium, as indicated, in the presence or absence of theophylline. For citrate-producing experiments, inocula were washed twice with sterile AD7 medium (3,000 *g*, 15 min, room temperature) and resuspended in sterile AD7 medium prior to inoculation. Citric acid production in cyanobacterial cultures was measured in the culture supernatants, following a centrifugation step (20,000 *g*, 5 min, room temperature) to remove cells and incubation of the cleared supernatant at 98°C, 5 min, to inactivate any possible remaining enzymatic activity. Cleared and heat-treated supernatants were frozen at −20°C until further use. For intracellular citric acid concentrations, 5 ml of the corresponding cultures were quenched in a liquid nitrogen/isopropanol bath for 20 s, centrifuged for 10 min at 5,000 *g*, 4°C, and the resulting pellet extracted with 10 ml of a water:methanol:chloroform mixture (1:3:6). After vortexing for 5 min, the extraction mixture was centrifuged for 5 min (5,000 *g*, room temperature), the aqueous phase collected, evaporated overnight at 80°C, and reconstituted in 500 μl of MQ water for quantification. Citric acid concentrations were measured in duplicate for each sample using the Citric Acid assay kit (K-CITR, Megazyme) in 96-well plates, following the manufacturer’s instructions.

## Results

### Citrate synthase overexpression does not increase citrate production

**Figure 3:**
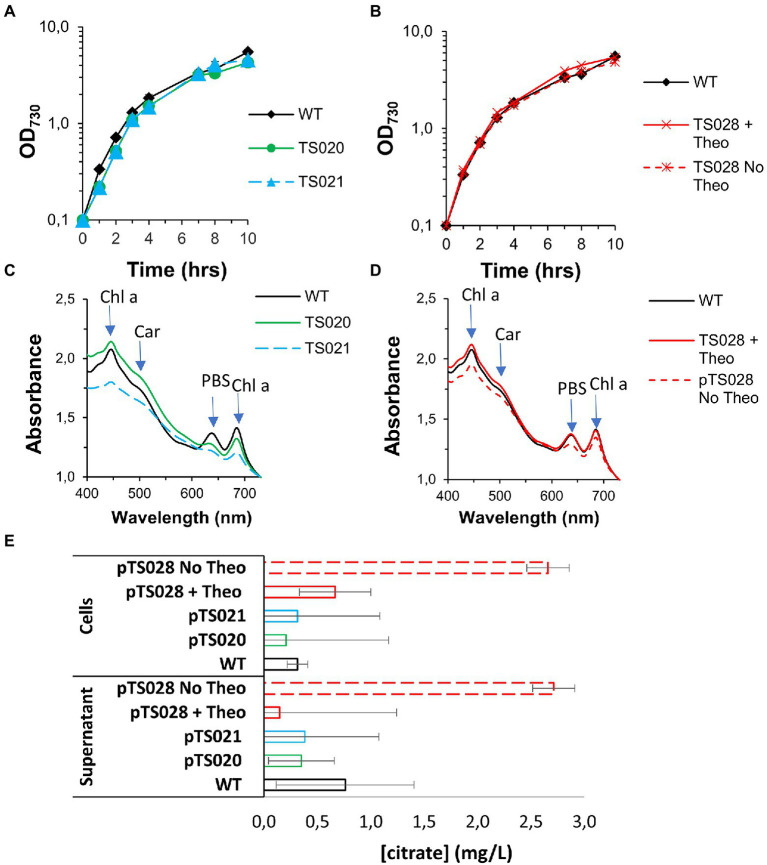
Growth curves and citrate quantification in AD7, air, low light conditions. **(A)** Growth curves for two citrate synthase overexpression strains. **(B)** Growth curves for strain TS028, without and with 2 mM theophylline (Theo) supplementation. **(C)** Whole cell spectra of WT, TS020 and TS021 at day 10. Arrows indicate absorbance maxima for chlorophyll a (Chl a), carotenoids (Car) and phycobilisomes (PBS). **(D)** Whole cell spectra of WT and TS028, without and with 2 mM Theo supplementation. **(E)** Citrate quantification in cell pellets and culture supernatants.

The TCA cycle occupies a central role in the metabolic network of aerobic organisms, with citrate synthases being highly regulated, both at the transcriptional and at the post-translational levels (Wiegand and Remington, 1986; Duckworth et al., 1987; Ito et al., 2019). At the same time, given that 2-oxoglutarate is a key metabolite for nitrogen assimilation, interruptions of the TCA cycle might have lethal consequences. To induce production of citric acid, we followed a “push-block-pull” strategy (Liu et al., 2018), generating overflow metabolism by improving flux toward the target product (“push”), blocking its consumption (“block”) and improving its secretion from the cell, to facilitate recovery (“pull”). We started by testing the effect of introducing four different citrate synthases to Syn7002, under control of a strong constitutive promoter (P_A2579_, Figure 2A; Ruffing et al., 2016). Surprisingly, we were unable to obtain strains expressing either the *E. coli* or the *S. coelicolor* citrate synthases, in spite of repeated attempts. It may be that either the activity or regulation of these enzymes causes a metabolic imbalance in Syn7002, rendering such strains unviable. Strains expressing Syn6803 (TS020) or *V. natriegens* (TS021) citrate synthases, on the other hand, were readily obtainable and stable (Figure 2B), though displaying mild growth defects and altered pigmentation in comparison to WT (Figures 3A,C). In both cases, phycobilisome content was decreased in comparison to WT, with TS020 (overexpressing Syn6803 citrate synthase) having an increased carotenoid content, in contrast to TS021, where carotenoid content was decreased. Chlorophyll a was reduced in both cases, more significantly in TS021 than in TS020 (Figure 3C).

Both strains were tested for citric acid production under air and low light conditions, and, in both cases, no citric acid accumulation could be detected, either in the supernatant or in the cell pellets, beyond the level detected for the WT (Figure 3E).

### Aconitase interruption is required for citrate accumulation and secretion

Citric acid produced by citrate synthase is exclusively converted to isocitrate by aconitase in Syn7002 (as a BLASTn search for citrate lyase genes found no homologs in the genome). Hence, metabolic flux control by reducing aconitase levels should result in accumulation of citric acid. However, given the possible lethality of the *acnB* deletion mutant, we generated a strain in which translation of this gene was controlled by the P_trc_-E* promoter (Chi et al., 2019), previously shown to allow tight control over target genes. Removing theophylline from culture medium should reduce AcnB levels in the cell due to protein turnover, resulting in a functional conditional knockdown. In line with this, transformants using pTS028 (Figure 2A) could be obtained and fully segregated only in the presence of both kanamycin and theophylline, as this allows translation of AcnB (Figure 2B).

Growth of strain TS028 in the presence of 2 mM theophylline (full induction conditions) was similar to that of Syn7002 WT (Figure 3B), with no change in pigmentation (Figure 3D). Surprisingly, culturing in the absence of theophylline resulted only in a mild growth defect under these conditions (Figure 3B), with reduced chlorophyll a, phycobilisome, and carotenoid content after 10 days of cultivation (Figure 3D).

While in the presence of theophylline, there was no significant intra/extracellular accumulation of citric acid beyond the levels observed in the WT, removing theophylline allowed an accumulation of citric acid in the cells, which could also be detected in the supernatant, to roughly 2.7 mg/L after 10 days of cultivation (Figure 3E).

### Combining aconitase depletion with citrate synthase overexpression improves citric acid production in high-density conditions

**Figure 4:**
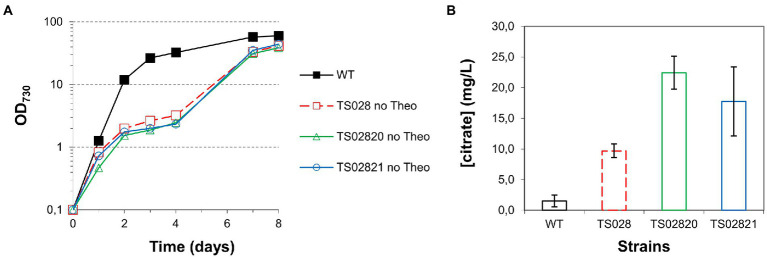
Growth curves and citrate quantification for first and second generation strains with *acnB* gene control. **(A)** Growth curves (in MAD, 1.5% CO_2_, high light). **(B)** Citrate quantification in medium at day 8.

Based on the results obtained from growth experiments in AD7, air and low light, growth and citric acid production of strain TS028 was tested in MAD, CO_2_-enriched air (1.5%) and high light (300 μmol photons·m^−2^·s^−1^), as these conditions were previously shown to allow faster growth of Syn7002 to high density and increase production of free fatty acids (Włodarczyk et al., 2020). Remarkably, a significantly more severe phenotype in high-density/fast growth conditions than that observed in air/low light became apparent in the absence of theophylline, with strain growth becoming arrested after 2 days (Figure 4A). After the 4th day, strain growth resumed, and cultures reached cell densities nearly as high as the WT cultures. Under these conditions, citric acid production was significantly improved, to roughly 10 mg/L.

To understand whether combined overexpression of citrate synthases and depletion of the aconitase could improve strain growth and/or citric acid production, we tested the second-generation strains TS02820 and TS02821 (with both the P_trc_-E* riboswitch in front of *acnB* and overexpression of Syn6803 or V. natriegens citrate synthases, respectively) under the same high-density conditions and in the absence of theophylline. In both cases, growth was severely disrupted, to a slightly larger extent than the TS028 strain, though, once more, strain growth resumed after the 4th day and final cell densities were similar to those of TS028 (Figure 4A). Concomitant expression of either of the citrate synthase genes resulted in a rough doubling of citric acid production in comparison to TS028, to nearly 20 mg/L (Figure 4B). Given the significant improvement to citric acid production in high-density/fast growing cultures, subsequent experiments were exclusively performed using MAD, CO_2_-enriched air and high light.

### Overexpression of the CexA exporter further improves citrate production

**Figure 5:**
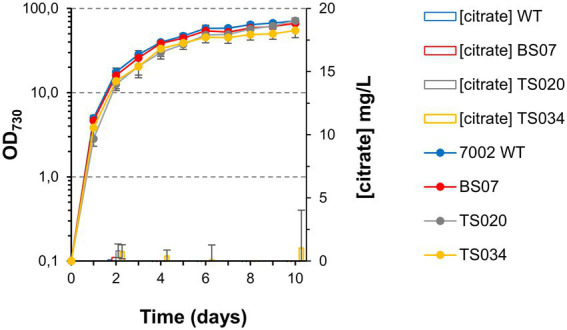
Growth curves and citrate quantification (in MAD, 1.5% CO_2_, high light) for background strains without *acnB* gene control.

Expression of the major *A. niger* citrate exporter protein was previously linked to improved citric acid production in *S. cerevisiae* (Steiger et al., 2019). We therefore tested whether combined overexpression of the CexA transporter with the Syn6803 citrate synthase in strain TS034 would be sufficient to induce citrate excretion from modified cells. However, as in the case of the simple citrate synthase overexpression, combined overexpression of the two proteins without aconitase control did not allow citric acid excretion beyond background strain levels (Figure 5).

**Figure 6:**
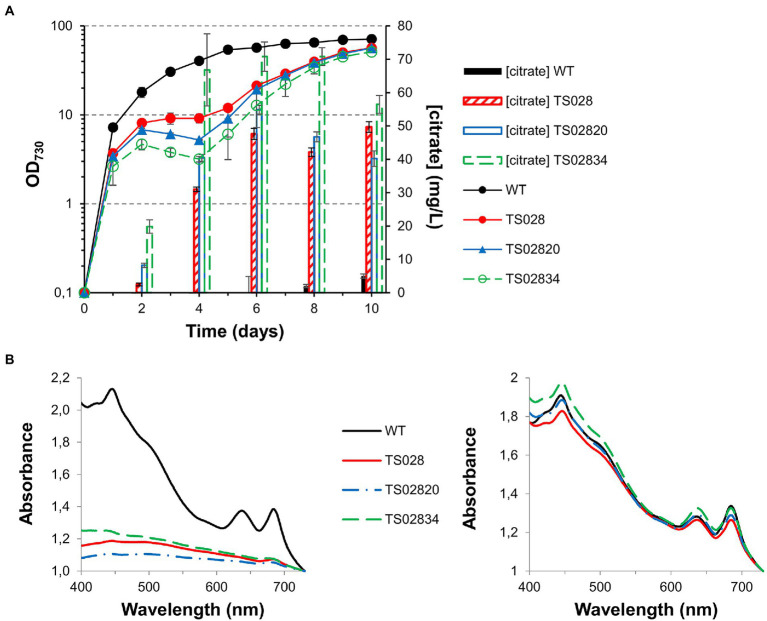
Growth curves, citrate quantification and whole cell spectra (in MAD, 1.5% CO_2_, high light) for first, second and third generation strains with *acnB* gene control. **(A)** Growth curves and citrate quantification. **(B)** Whole cell spectra at day 3 (left) and day 8 (right).

Given the results obtained with the second-generation strain TS02820, a third-generation strain, combining aconitase control with citrate synthase and CexA overexpression, was tested alongside its earlier iterations. While introduction of CexA led to the growth of this strain (TS02834) being more severely affected after the second day (in the absence of theophylline) and growth recovery taking longer than in the case of TS02820 or TS028, its production titer was significantly improved in the first 6 days, reaching 70 mg/L under high-density conditions (Figure 6A). In all cases, the defect on strain growth was accompanied by a severe bleaching phenotype, followed by a recovery of cell pigmentation toward the end of the experiment (Figure 6B), which could be indicative of genetic instability of these strains under conditions of high metabolic burden.

### Glutamate supplementation relieves growth inhibition during citrate production

The introduction of the P_trc_-E* riboswitch combination allows for metabolic flux through the TCA cycle to be restricted “on demand,” with concomitant citrate production in TS02834. However, this came at the cost of a severe disruption to growth and pigmentation. Interrupting the TCA cycle disrupts 2-oxoglutarate production, a key metabolite for nitrogen assimilation into glutamate, either through the glutamine synthetase/glutamine-oxoglutarate aminotransferase (GS/GOGAT) cycle or glutamate dehydrogenase activities (Flores and Herrero, 2005). We therefore hypothesized that supplementing cultures with glutamate could be sufficient to relieve growth inhibition during citric acid production.

**Figure 7:**
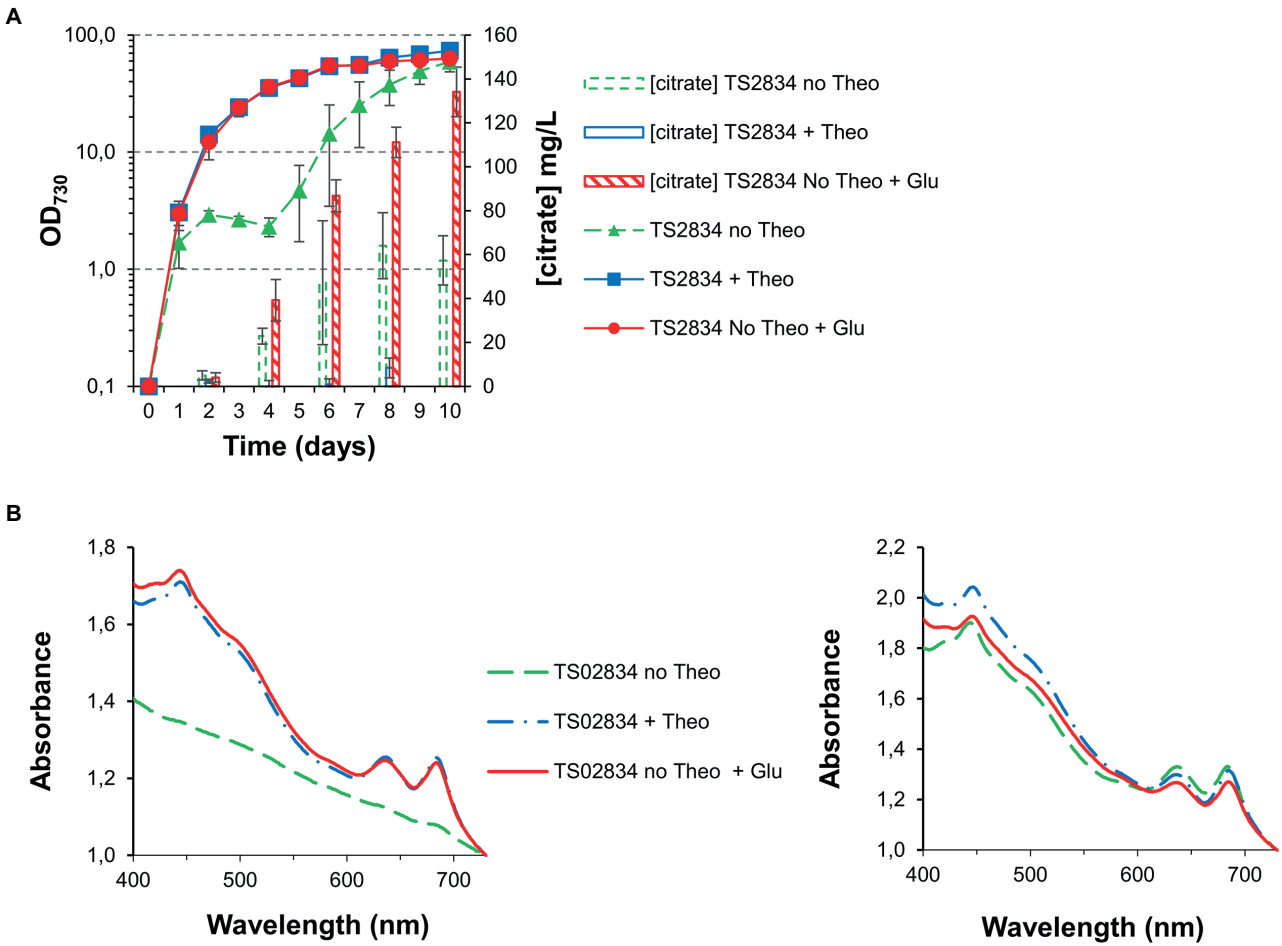
Growth curves, citrate quantification and whole cell spectra (in MAD, 1.5% CO_2_, high light) for third generation strains with *acnB* gene control, without and with glutamate supplementation. **(A)** Growth curves and citrate quantification. **(B)** Whole cell spectra at day 3 (left) and day 8 (right).

Growing TS02834 in the presence of an excess of glutamate (50 mM) allowed both cell growth and pigmentation to remain similar to that observed for cultures grown in the presence of full theophylline induction (2 mM; Figures 7A,B): While cells cultured in the absence of theophylline displayed the “S-shaped” growth curve previously observed, this growth arrest was completely absent in glutamate-treated cells (Figure 7A). Moreover, glutamate supplementation allowed TS02834 citric acid production to remain unabated during the whole course of the growth experiment, resulting in an almost doubling of the production titer, to roughly 130 mg/L after 10 days.

### Riboswitch titration balances citrate production and strain growth

**Figure 8:**
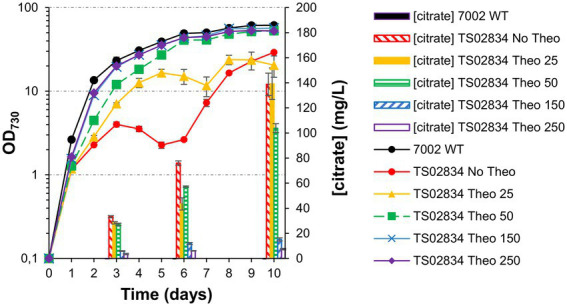
Growth curves, citrate quantification and whole cell spectra (in MAD, 1.5% CO_2_, high light) for TS02834 strain 3, with varying amounts of theophylline (in µmol). **(A)** Growth curves and citrate quantification. **(B)** Whole cell spectra at day 3 (left) and day 8 (right).

Given the intrinsic variability between TS02834 biological triplicates chosen (Supplementary Figure S2), strain TS02834-3 (the highest producing strain) was chosen to test whether a balance between aconitase expression level and citrate production could be achieved by titrating theophylline levels in the culture. Culturing in the presence of varying levels of theophylline modulated the growth phenotype severity. As a consequence, citric acid titers also varied, with cells cultured in the absence of theophylline being the most significantly affected (Figure 8). While maximum titers in the presence of 25 μM theophylline (“Theo25”) were similar to those of cells grown in absence of theophylline (“No Theo”; 139.3 vs. 138.8 mg/L, respectively), growth was still very severely affected, even if the cultures could initially outperform the “no theophylline” control. Cells grown in the presence of 50 μM theophylline (“Theo50”) were able to attain much higher cell densities (OD_730_ = 52.9 for “Theo50” vs. OD_730_ = 20.6 for “Theo25”), nearly as high as the WT control, though with a *ca.* 25% reduction in citric acid production. At concentrations above 150 μM theophylline, cell growth was unaffected though citric acid production was minimized.

We hypothesized that the recovery from growth arrest observed for citric acid-producing cultures (in the absence of theophylline) could be due to mutations arising in the riboswitch aptamer region, which could lead to repression of aconitase being lost. We therefore sequenced the promoter region of *acnB* in cultures grown in the presence/absence of theophylline and in the absence of theophylline with glutamate supplementation (Supplementary Figure S1). We found that the metabolic burden caused by severely constricting flux through aconitase did, as we previously speculated, induce mutations in the theophylline aptamer region in cultures grown in the absence of theophylline and glutamate.

## Discussion

Current citric acid production is dominated by fermentative production using *Aspergillus* strains grown on different carbon sources (Xue et al., 2021). This work presents a first proof-of-concept that engineered cyanobacteria are able to convert CO_2_ to citrate, even if production titers are currently low in comparison to published data for modified *Aspergillus* strains [174.1 g/L, (Xue et al., 2021)]. While cyanobacterial citrate production is for the moment unlikely to supply the large amounts required by, e.g., the food and beverage industry in the near future, improving productivity could lead to an important route for closing production loops in other industries, by recovering CO_2_ from process gas waste streams (e.g., from hydrometallurgical battery recycling) and converting it into citric acid, which could then be reused in the same process. Similarly, an improved version of this process could contribute to lower the carbon footprint of fermentative citric acid production itself, by converting carbon lost as CO_2_ during the growth of *Aspergillus* cultures back to citric acid.

Citric acid plays a central role in cellular metabolism, as the first intermediate in the TCA cycle, and is produced through a single enzymatic reaction (the condensation of acetyl-CoA and oxaloacetate by citrate synthase). In cyanobacteria, the TCA cycle is a source of key metabolites, linking carbon fixation and nitrogen assimilation through one of its intermediates, 2-oxoglutarate (Espinosa et al., 2006). In these organisms, the TCA cycle is characterized by the lack of a 2-oxoglutarate dehydrogenase complex, with succinate synthesis occurring through the action of 2-oxoglutarate decarboxylase and succinate semialdehyde dehydrogenase (Zhang and Bryant, 2011). Though the metabolic flux through this shunt is rather low (Hendry et al., 2016) TCA cycle-derived 2-oxoglutarate is critical for nitrogen assimilation and biomass production. Thus, in order to use cyanobacteria as a production platform for citric acid, a calibrated approach had to be devised.

As shown in Figures 3, 6, a simple overexpression of citrate synthase, even with a strong constitutive promoter (“push”), is not sufficient to generate enough metabolic overflow to citric acid and enable either its accumulation in the cell or excretion to the growth medium. The cyanobacterial aconitase, as recently shown for the Syn6803 enzyme, has a K_m_ for citric acid of 1.1 mM (Nishii et al., 2021). While 10-fold lower than the corresponding E. coli counterpart, the flux through aconitase was seemingly enough to accommodate an increase in the flux through the citrate synthase node, even when different citrate synthases were overexpressed, resulting in no significant citric acid accumulation.

Reducing flux through the TCA cycle at the level of aconitase (“block”) was shown to be sufficient to induce citric acid accumulation and excretion (Figure 3). Improving the flux through citrate synthase (to increase the available pool of citric acid) in combination with a blockage of its consumption (through a decrease in aconitase expression) resulted in a significant increase in citrate production. Furthermore, this improvement could be enhanced by the introduction of a “pull” force, in the form of the citrate exporter CexA. However, while introducing the CexA exporter was previously shown to improve citric acid excretion in yeast cells (Steiger et al., 2019), this was insufficient to drive citric acid excretion from TS034 cells, which also overexpress Syn6803 citrate synthase. This could be due to its quick conversion to isocitrate by aconitase, underscoring the need to reduce citrate consumption by the aconitase reaction in order to improve citrate accumulation and excretion.

Interestingly, knocking down aconitase expression did not affect TS028 strain growth as significantly in ambient CO_2_/low light as it did in elevated CO_2_ and high light intensity conditions (Figures 3B, 4A). It may be that in low light and ambient CO_2_, the flow through the TCA cycle and the requirement of 2-oxoglutarate for nitrogen assimilation, while remaining of critical importance, could be matched by the relatively slower growth of Syn7002. Alternatively, either aconitase turnover is slower in those conditions or the low-level leakiness from the P_trc_-E* uninduced promoter is sufficient under these slower growth conditions. Even if phycobilisome degradation could be observed to some extent, the effect was not as severe as when TS028 and related strains were cultured in high-density conditions (vd. Figures 3, 4). Under elevated CO_2_/high light, metabolic flux through the TCA cycle (given the faster cell growth under these conditions) is likely to be higher than when cells are cultured in ambient CO_2_/low light, as increased cell division rates are likely to be linked to a higher demand for protein synthesis and, inherently, nitrogen assimilation/amino acid biosynthesis. This higher pressure on 2-oxoglutarate—and consequently, glutamate—synthesis in faster growing cells (in high-density conditions) may then lead to a much more rapid degradation of cellular proteins, such as phycobiliproteins and photosystems, than in ambient CO_2_/low light, in a bid to maintain amino acid levels and cellular functionality. A similar effect (pigment degradation and chlorosis) was also previously reported when citrate synthase expression was blocked by CRISPRi in a lactate-producing strain (Yao et al., 2020), with glutamate supplementation also being able to relieve growth arrest under those conditions, in line with our current results. This higher pressure in faster-dividing cultures is likely the causative agent of the observed mutations in the theophylline riboswitch (Supplementary Figure S1). The restricted flux through aconitase in fast-growing conditions (MAD and high CO_2_) resulted in the stunted growth and decreased pigmentation phenotypes observed. The induced stress likely creates a selective pressure that favors mutations allowing the riboswitch to relax. Indeed, the secondary structure prediction using Unafold (Markham and Zuker, 2008; Supplementary Figure S1) suggests these mutations may expose part of the ribosome binding site and the start codon (previously sequestered by the riboswitch’s stem-loop structure), which could then lead to increased leaky expression of aconitase and resumption of TCA cycle activity. These mutations were not observed in cultures grown without theophylline but supplemented with glutamate (as supplementation with this amino acid most likely relieves metabolic constraints in the strain) or at theophylline concentrations higher than 25 μM (data not shown). As these mutations likely permit aconitase expression to be resumed (to a level that is sufficient to support robust nitrogen assimilation and cell growth), normal growth and pigmentation are recovered, at the cost of decreased production.

Recently, Shabestary and co-workers (Yao et al., 2020; Shabestary et al., 2021) showed that the CRISPRi-induced repression of citrate synthase improved lactate production and that cycling between growth arrest and biomass accumulation (by continuously diluting the culture and cyclically adding anhydrotetracycline to repress gene expression) resulted in strain growth and lactate production improvement. A similar strategy could be adopted in the future for citric acid production, by linking the expression of dCas9 to a synthetic oscillator circuit, as previously suggested (Shabestary et al., 2021). While this could in theory be applied to the current strains, we have noticed that culturing strain TS02834 to higher cell densities (OD_730_ ≈ 10) before removing theophylline did not result in citric acid production (data not shown). Flux through the TCA cycle (and aconitase) could have a more critical importance in the early growth phase or, at higher cell densities, turnover of the remaining aconitase might be slower, negating the control afforded by the riboswitch. The current strategy initiates citrate production in the absence of the chemical inducer theophylline, allowing control over TCA cycle flux and biomass accumulation (for cultivating stock cultures for inoculation) while lowering production costs at scale (by the absence of the chemical inducer in producing cultures). Our engineering strategy (using a titratable riboswitch to control aconitase expression) allowed us to probe the effect of dynamically restricting metabolic flux at the level of this enzyme. Our data demonstrate that appropriately balancing flux through aconitase (Figure 8) leads to sustained citric acid production and improved strain stability. While the theophylline riboswitch provides a robust solution for dynamic flux control in proof-of-concept studies such as this one, it would be economically unviable to use such a control system in a scaled-up process. Further iterations of the current strains could test constitutive promoter libraries (Markley et al., 2015) to express aconitase at the right level to balance these two constraints. Alternatively, a more dynamic control system—for instance, by integrating both 2-oxoglutarate and citrate levels through a NAND gate (Lee and Woo, 2020) to control aconitase output—could prove more useful than a constitutive promoter, by continuously adapting flux restriction to cellular metabolic requirements. Additionally, future strain iterations should also aim to improve flux toward oxaloacetate and/or acetyl-CoA as this may also increase production titers, by improving the available pools of both precursors.

In conclusion, this work demonstrates that conversion of CO_2_ (which could be derived from industrial waste gases) to citric acid using modified cyanobacteria is possible. While further strain engineering is still required, for improved production titers and strain growth, our results are a first step toward sustainable production of one of the world’s most utilized organic acids.

## Data availability statement

The original contributions presented in the study are included in the article/Supplementary material, further inquiries can be directed to the corresponding authors.

## Author contributions

LZ, SB, and TS devised the experiments, analyzed the data, and wrote the manuscript. LZ and TS performed the experiments. All authors contributed to the article and approved the submitted version.

## Funding

This work was funded by UKRI through the Carbon Recycling Network, grant number BIV-03-BRYAN-CCNet, as a part of BB/S009833/1.

## Conflict of interest

The authors declare that the research was conducted in the absence of any commercial or financial relationships that could be construed as a potential conflict of interest.

## Publisher’s note

All claims expressed in this article are solely those of the authors and do not necessarily represent those of their affiliated organizations, or those of the publisher, the editors and the reviewers. Any product that may be evaluated in this article, or claim that may be made by its manufacturer, is not guaranteed or endorsed by the publisher.

## Supplementary material

SUPPLEMENTARY FIGURE S1 Induced mutations on theophylline aptamer. **(A)** Alignment of TS02834 theophylline riboswitch region after 10 days culturing in the absence of theophylline, without or with glutamate supplementation. **(B)** Secondary mRNA structure of original (left) and mutated (right) theophylline riboswitch aptamer. Structures simulated using the mFold tool in OligoAnalyzer package. Red lines mark RBS sequence.

SUPPLEMENTARY FIGURE S2 Biological variability in citrate production between citrate producing strains. **(A)** Citrate production in three independent production strains in the absence of theophylline and glutamate supplementation. **(B)** Citrate production in three independent production strains in the absence of theophylline and no glutamate supplementation.

SUPPLEMENTARY TABLE S1 List of primers used in this work.
