# Supplementary figures and images for: Sustainable citric acid production from CO2 in an engineered cyanobacterium
Source: Front Microbiol. 2022 Aug 17;13:973244. doi: 10.3389/fmicb.2022.973244 (PMC9428468; doi:10.3389/fmicb.2022.973244)

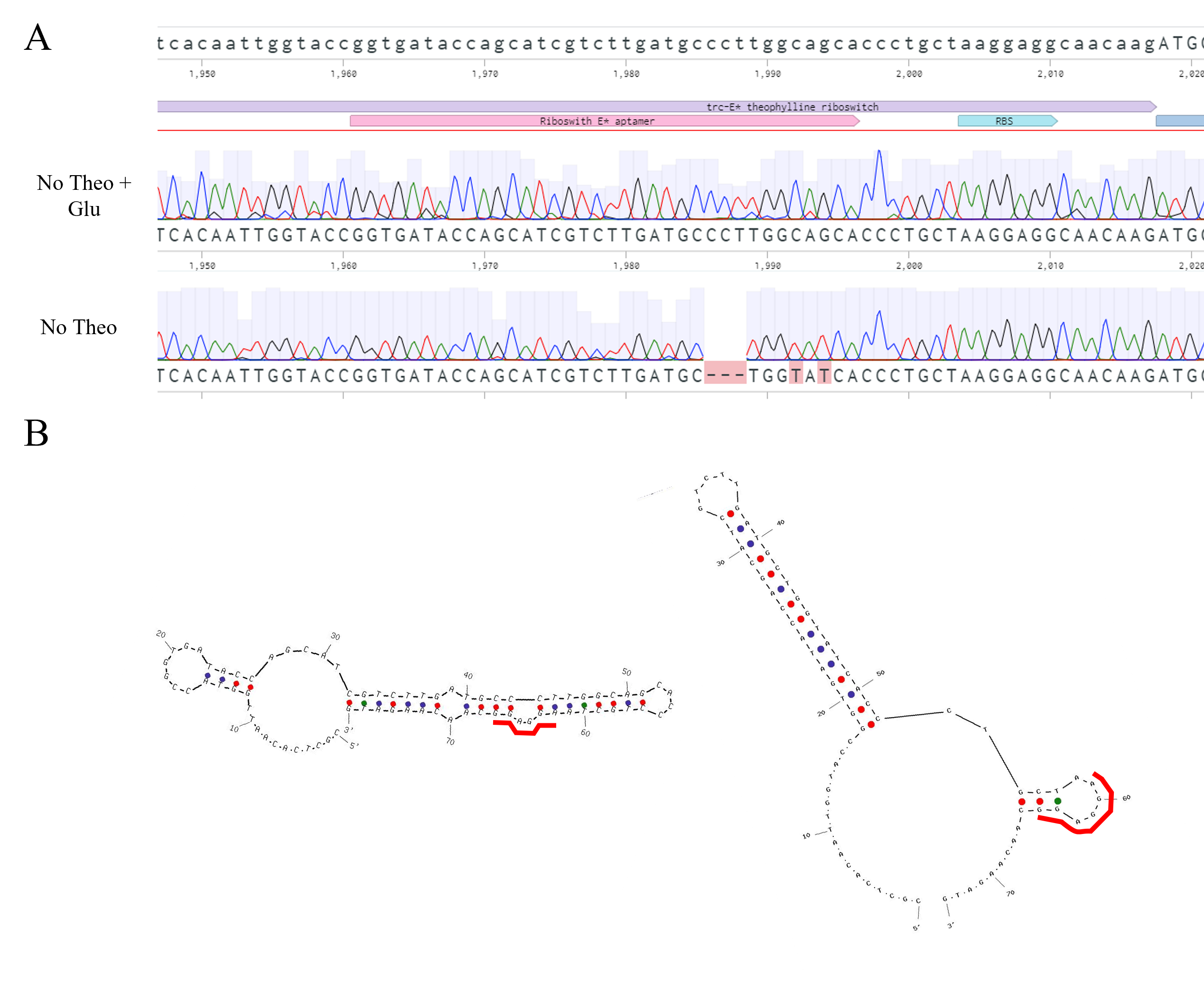

Supplement: SUPPLEMENTARY FIGURE S1 — Induced mutations on theophylline aptamer. (A) Alignment of TS02834 theophylline riboswitch region after 10 days culturing in the absence of theophylline, without or with glutamate supplementation. (B) Secondary mRNA structure of original (left) and mutated (right) theophylline riboswitch aptamer. Structures simulated using the mFold tool in OligoAnalyzer package. Red lines mark RBS sequence. [file Image_1.TIF]

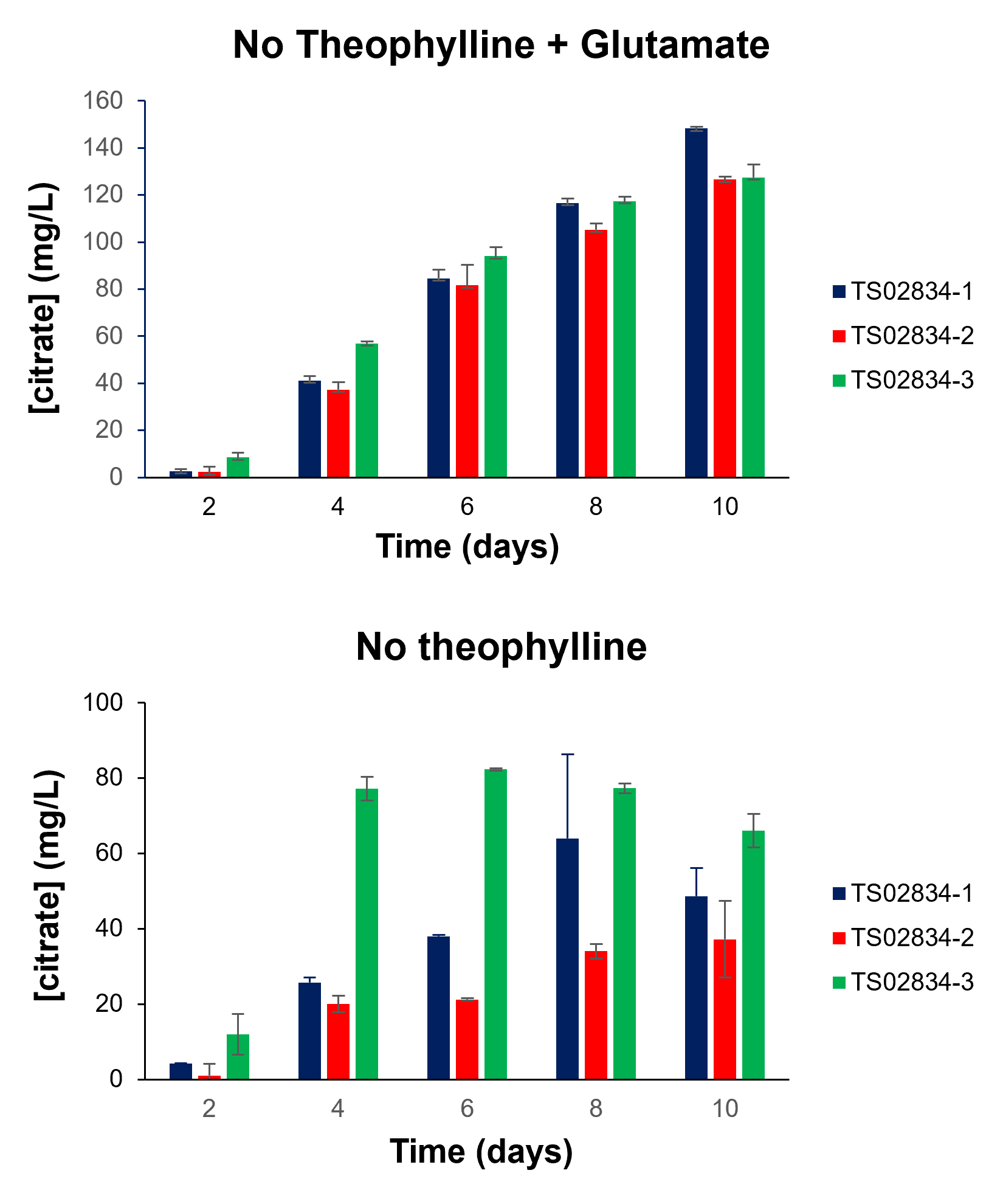

Supplement: SUPPLEMENTARY FIGURE S2 — Biological variability in citrate production between citrate producing strains. (A) Citrate production in three independent production strains in the absence of theophylline and glutamate supplementation. (B) Citrate production in three independent production strains in the absence of theophylline and no glutamate supplementation. [file Image_2.TIF]
